# Prevalence and risk factors of frailty in patients with gynecological malignancies after surgery and chemotherapy: a cross-sectional study

**DOI:** 10.3389/fonc.2025.1629212

**Published:** 2026-01-09

**Authors:** Tingting Wang, Zihe Song, Xianliang Liu, Xia Duan

**Affiliations:** 1 Nursing Department, Shanghai Key Laboratory of Maternal Fetal Medicine, Shanghai Institute of Maternal-Fetal Medicine and Gynecologic Oncology, Shanghai First Maternity and Infant Hospital, School of Medicine, Tongji University, Shanghai, China; 2 Department of Cardiology, The Third Hospital of Hebei Medical University, Shijiazhuang, China; 3 School of Nursing and Health Sciences, Hong Kong Metropolitan University, Hong Kong, Hong Kong SAR, China

**Keywords:** chemotherapy, cross-sectional study, frailty, gynecological oncology, surgery

## Abstract

**Purpose:**

Frailty is a significant factor influencing the prognosis of patients with gynecological malignancies. Surgery and chemotherapy are key components of cancer treatment; however, limited research has addressed frailty and its associated risk factors in this patient population. This study aimed to determine the prevalence of frailty and identify its associated factors in patients with gynecological malignancies following surgery and chemotherapy.

**Method:**

This cross-sectional study was conducted at two tertiary hospitals in Shanghai between January and September 2024. Patients completed five assessment scales, including the Demographic Information Scale, Vulnerable Elders Survey-13 (VES-13), Athens Insomnia Scale (AIS), Depression Anxiety Stress Scale-21 (DASS-21), and Perceived Social Support Scale (PSSS), to screen for frailty and related factors. Multivariate logistic regression analysis was used to identify risk factors for frailty, with 95% confidence intervals (CIs) used to estimate the strength of the associations.

**Result:**

A total of 211 eligible inpatients with gynecological malignancies who had undergone surgery and chemotherapy were recruited. The prevalence of frailty in these patients was 64.9%. Multivariate logistic regression analysis identified several risk factors for frailty: older age, underweight status, stress, insomnia, married status, high white blood cell count, and low albumin levels.

**Conclusion:**

This study highlights the relatively high prevalence of frailty among patients with gynecological malignancies after surgery and chemotherapy. Understanding the factors associated with frailty is crucial for implementing timely interventions that can improve health-related quality of life and treatment outcomes in this patient population.

## 1 Introduction

Gynecological malignancies, including ovarian, cervical, and endometrial cancers, are prevalent among adult women of all age groups (1). According to data from the World Health Organization (WHO) in 2020 (2), the global incidence of ovarian, cervical, and endometrial cancers exceeded 1.33 million cases, accounting for 14.4% of all malignant tumors in women, with an associated mortality rate of approximately 550,000. In China, there were approximately 170,000 new cases of these three major gynecological malignancies, resulting in nearly 100,000 deaths and accounting for 35.38% of the global total (3). These statistics highlight the significant public health challenges posed by gynecological malignancies, which threaten the lives and health of women worldwide.

Surgery and chemotherapy are the main treatment methods for tumors (4, 5), aiming to remove tumor lesions and inhibit the spread of cancer cells to improve survival opportunities for patients. However, research indicates that despite advances in medical technology and longer survival, 66% of the more than 15 million patients with cancer living in the United States currently have at least one other chronic health condition (6). It has become increasingly evident that patients undergoing surgery and chemotherapy face many complicated and long-lasting health problems.

Frailty is a multidimensional syndrome characterized by organ dysfunction, reduced physiological reserves, heightened vulnerability, and diminished resilience to stress. It has garnered increasing attention in oncology research (7, 8). A systematic review revealed that the prevalence of frailty in patients with gynecological malignancies ranges from 6.1% to 47.9% (9–11), significantly higher than the average frailty prevalence of 34% in patients with cancer overall (12). Furthermore, frailty has been identified as a key factor affecting the overall quality of life in both postoperative and chemotherapy patient populations, including increased postoperative complications (13), chemotherapy-related toxicity (14), prolonged hospital stay (15), higher readmission rates, and mortality (16). These findings highlight the importance of understanding frailty in patients with gynecological cancer to provide early interventions that may improve patient outcomes and quality of life.

Although several studies have explored the determinants of frailty in patients with cancer, most have focused on specific subgroups or treatment stages. For instance, Ding et al. (17) found in their study of 408 patients with gastric cancer that nutrition, anemia, and comorbidities were factors influencing preoperative frailty. Similarly, Jeon et al. (18) discovered that depression and heart rate variability were factors influencing frailty in patients with cancer undergoing chemotherapy. However, few studies have simultaneously considered frailty in patients with gynecological cancer following surgery and chemotherapy. Given the critical role of frailty on clinical outcomes, this gap in the literature warrants further investigation. This study aimed to address this research gap by examining the prevalence of frailty and identifying its associated factors in patients with gynecological malignancies following surgery and chemotherapy. The findings are intended to inform the development of targeted interventions to improve the health and well-being of this vulnerable patient population.

## 2 Methods

### 2.1 Sample and setting

This cross-sectional study used convenience sampling to select patients with gynecological malignancies who received treatment at two Grade III hospitals in Shanghai between January and September 2024.

Inclusion criteria:

1. First-time diagnosis of cervical, ovarian, or endometrial cancer based on clinical and pathological evidence, with subsequent adjuvant chemotherapy following surgery.
2. Aged ≥18 years
3. Provision of informed consent and voluntary participation in the study.

Patients were excluded if they (1) had mental disorders that hindered normal cooperation or communication barriers (e.g., schizophrenia, attention deficit hyperactivity disorder) (2), had a history of serious systemic diseases or other malignancies (e.g., lung cancer, AIDS), or (3) received neoadjuvant chemotherapy prior to surgery, or (4) not receive adjuvant chemotherapy after the surgery.

### 2.2 Procedures

After obtaining ethical approval, two researchers underwent standardized training to identify potentially eligible participants using electronic medical records. Participants were then approached and provided with a detailed explanation of the purpose and procedures of the study. This ensured that potential participants fully understood the study before making informed decisions and providing written consent. After obtaining written consent, the research team collected data on participants’ frailty, sleep quality, social support, anxiety, depression, and stress levels using an online questionnaire. For participants unable to complete the questionnaire independently, the researchers administered it using neutral and non-directive language to ask questions for each item on the scale and accurately recorded the patients’ responses. Only fully completed questionnaires were deemed valid for the data analysis.

### 2.3 Measurement

#### 2.3.1 Demographic information

A demographic information questionnaire was developed based on a review of the literature (19, 20). It included two sections: demographic data (e.g., age, marital status, education level, occupation, income, and medical and disease history) and clinical data (e.g., pathological classification, tumor stage, comorbidities, and chemotherapy regimen).

#### 2.3.2 Vulnerable elders survey-13

Frailty was assessed using the Vulnerable Elders Survey-13 (VES-13) questionnaire, a validated self-assessment tool developed by Saliba et al. in 2001 (21) to screen for frailty. For this study, the Chinese version of the VES-13, translated and validated by Wu et al. (22), was used. The questionnaire consisted of 13 items covering four domains: age, self-assessment of health, activity status, and functional status. The total VES-13 score ranges from 0 to 10, with scores >3 indicating frailty. Higher scores reflect greater frailty severity. The VES-13 has been widely applied in screening for frailty among patients with cancer (23, 24). In this study, the VES-13 demonstrated good internal consistency, with Cronbach’s α coefficient of 0.782.

#### 2.3.3 Athens insomnia scale

The Athens Insomnia Scale (AIS) is a standard tool for assessing sleep difficulties based on the ICD-10 classification (25). The scale consists of eight items with a total score ranging from 0 to 24. Higher scores indicate more severe insomnia; scores <4 indicate no disorder, 4–6 indicate possible sleep disturbances, and >6 indicate insomnia. The Cronbach’s α coefficient of the AIS scale in this study was 0.905.

#### 2.3.4 The depression anxiety stress scale-21

The Depression Anxiety Stress Scale-21 (DASS-21) is a self-rating scale developed to assess the severity of negative emotional symptoms with 21 items covering depression, anxiety, and stress. This was adapted from the original 42-item scale developed by Antony et al. (26, 27). A 4-point scale ranging from 0 (non-conformity) to 3 (most conformity) was used. The sum of the scores of each subscale item multiplied by 2 was the subscale score. The scores ranged from 0 to 42 points. In this study, the Cronbach’s α coefficient of the DASS-21 total scale was 0.896, and the Cronbach’s α coefficient of the depression, anxiety, and stress subscales was 0.896, 0.766, and 0.821, respectively.

#### 2.3.5 Perceived social support scale

Social support was assessed using the Perceived Social Support Scale (PSSS) questionnaire, developed by Dahlem and Zimet in 1987 (28), which consists of 12 items evaluating two dimensions: family support and external support. Each question has seven options, ranging from “strongly disagree” to “strongly agree,” using a 7-point Likert scale where the total score is calculated by adding the scores for all 12 items, with a score of 12–36 being low support, 37–60 being medium support, and 61–84 being high support. The higher the score, the greater the social support felt. In this study, the PSSS demonstrated excellent internal consistency, with a Cronbach’s α coefficient of 0.974.

### 2.4 Statistical analysis

All analyses were performed using IBM SPSS Statistics for Windows, version 24.0. Continuous variables with a normal distribution (e.g., age, red blood cell count, white blood cell count) were presented as means ± standard deviation (SD), and categorical variables (e.g., birthplace, ethnicity, marital status) were expressed as frequency and percentage (N (%)). Correlations between sleep, social support, anxiety, depression, stress, and frailty scores were analyzed using Spearman’s correlation analysis. Independent t-tests were used to compare continuous variables between groups, whereas chi-squared tests were applied to categorical variables to assess differences between patients with gynecological malignancies. Additionally, binary logistic regression was used to identify factors associated with frailty. The results were reported as odds ratios (ORs) with 95% confidence intervals (CIs). All p-values were two-tailed, and *p ≤*0.05 was considered statistically significant.

### 2.5 Ethics considerations

This study was conducted in accordance with the principles of the Declaration of Helsinki. Ethical approval was obtained from the hospital’s Ethics Committee (KS232B8). Participation was entirely voluntary, and individuals were informed of their right to withdraw from the study at any time without any repercussions. To protect confidentiality, all personal data were anonymized, and participant records were securely stored in accordance with data protection regulations.

## 3 Results

**Figure 1:**
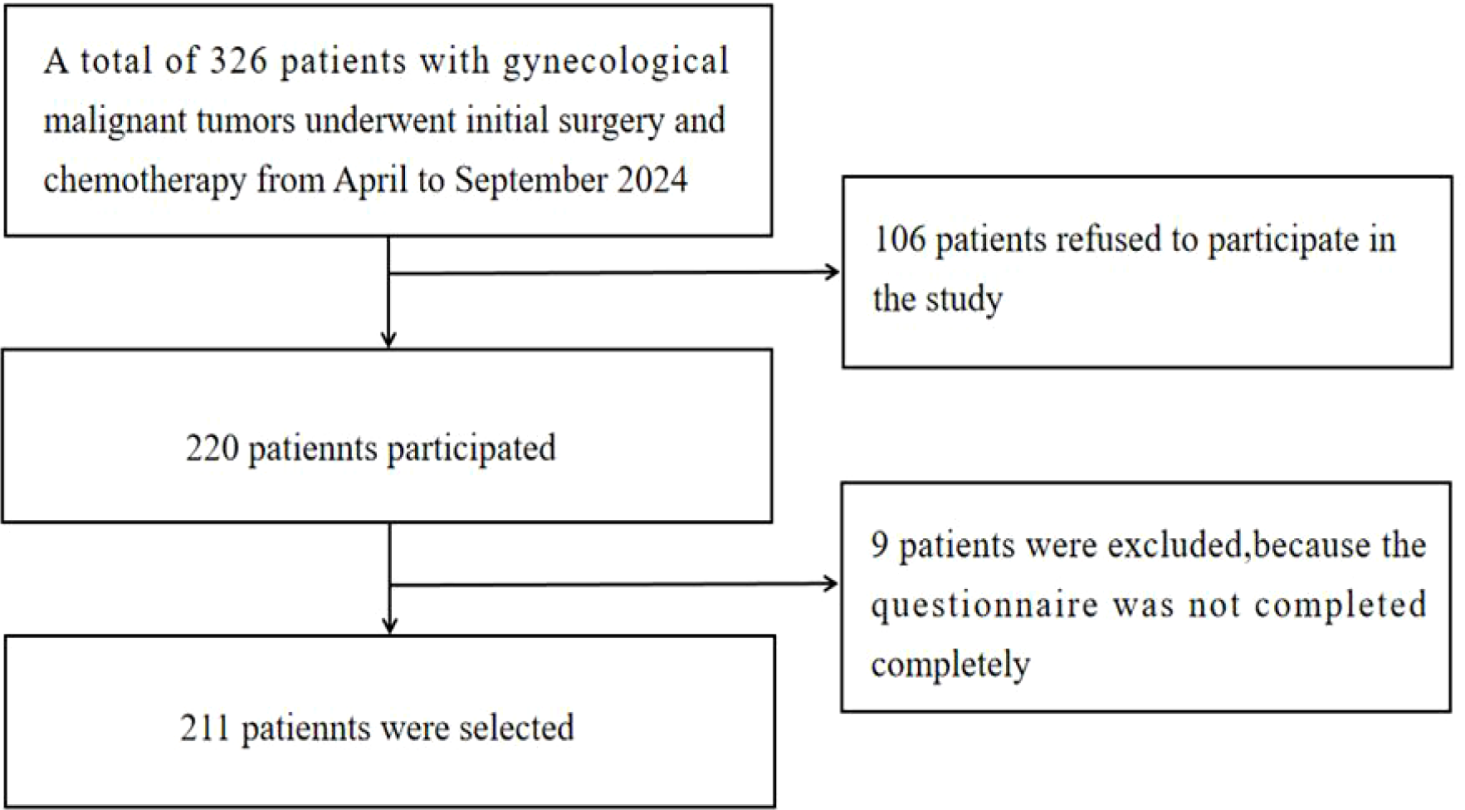
Flow chart of participants.

During the survey, 326 patients met the inclusion criteria. Of these, 220 patients expressed willingness to participate in the survey and signed an informed consent form. We excluded data from nine patients who did not complete the questionnaire. Thus, data from 211 patients were analyzed (**Figure 1**).

**Tables 1**, **2** summarize the demographic and clinical characteristics of the participants, stratified by frailty status. The mean age of the participants was 54.98 years. Of the total participants, 137 (64.9%) were classified as frail, while 74 were classified as non-frail. Univariate analysis identified several variables with significant differences between the two groups. These included age, marital status, occupation, body mass index (BMI), poor lifestyle habits, tumor stage, therapy method, combined chronic diseases, personal monthly income, sleep quality, social support, depression, stress, white blood cell count, and albumin levels. These variables were identified as potential risk factors for frailty in patients with gynecological cancer undergoing surgery and chemotherapy.

**Table 1:** Demographic and clinical characteristics of participants by frailty group.

Variables	Total (N = 211)	Non-frail(N = 74)	Frailty (N = 137)	Statistics	p-value
Age	54.98 ± 11.226	48.07 ± 9.973	58.71 ± 10.061	F=54.070	<0.001
Birthplace				χ²=1.081	0.299
Shanghai	90	28	62		
Non-Shanghai	121	46	75		
Nation				χ²=0.182	0.670
Han nationality	207	73	134		
Else	4	1	3		
Marital status				χ²=9.560	0.023
Married	179	59	120		
Unmarried	9	6	3		
Divorced	14	8	6		
Widowed	9	1	8		
Resident manner				χ²=3.792	0.285
Solitary	24	11	13		
Spouse only	91	27	64		
More than two generations together	93	34	59		
Live with others	3	2	1		
Degree of education				χ²=3.758	0.153
Junior high school and below	93	27	66		
High school or technical secondary school	67	24	43		
College degree or above	51	23	28		
Occupation				χ²=13.949	0.016
Civil servant	4	2	2		
Company employee	51	28	23		
Self-employed	24	8	16		
Worker/Farmer	15	5	10		
Unemployed	13	5	8		
Retirement	104	26	78		
Mode of payment				χ²=6.644	0.084
Urban residents insurance	69	22	47		
Employee medical insurance	107	42	65		
New rural cooperative	33	8	25		
Self-financing	2	2	0		
BMI					
Underweight (< 18.5)	35	10	25		
Normal (18.5–24.9)	116	62	54		
Overweight (> 24.9)	60	2	58		
Unhealthy lifestyle habits				χ²=12.085	<0.001
No	155	65	90		
Smoking/Alcohol/Inactivity	56	9	47		
Cancer type				χ²=0.067	0.967
Cervical cancer	90	31	59		
Ovarian cancer	69	24	45		
Endometrial cancer	52	19	33		
Tumor stage				χ²=12.860	0.005
Stage I	62	31	31		
Stage II	35	9	26		
Stage III	99	33	66		
Stage IV	15	1	14		
Therapy method				χ²=7.234	0.007
Only chemotherapy	125	53	72		
With other treatments (such as radiotherapy, immunotherapy, etc.)	86	21	65		
Combined chronic diseases				χ²=9.644	0.002
Yes	75	16	59		
No	136	58	78		
Personal monthly income				χ²=11.050	0.004
Less than 3000 yuan	61	11	50		
3000 to 7000 yuan	88	36	52		
More than 7000 yuan	62	27	35		

BMI, body mass index.

**Table 2:** Level of participants’ sleep, fatigue, social support, anxiety, depression, stress, and blood indicators data by frailty group.

Variables	Total (N = 211), Mean ± SD	Non-frail (N = 74), Mean ± SD	Frailty(N = 137), Mean ± SD	Statistics	P-value
Sleep (AIS)	4.50 ± 4.439	2.41 ± 3.05	5.62 ± 4.75	F=27.460	<0.001
Social support (PSSS)	59.44 ± 10.63	60.08 ± 7.72	59.09 ± 11.92	F=0.412	0.521
Anxiety (DASS-21)	5.63 ± 3.08	3.87 ± 3.15	6.57 ± 2.60	F=44.191	<0.001
Depress (DASS-21)	8.73 ± 5.73	4.58 ± 4.72	10.97 ± 4.92	F=83.267	<0.001
Stress (DASS-21)	7.25 ± 4.19	3.68 ± 3.25	9.18 ± 3.28	F=136.394	<0.001
White blood count	6.78 ± 2.45	5.62 ± 1.84	7.41 ± 2.52	F=28.781	<0.001
Red blood count	4.21 ± 0.66	4.28 ± 0.75	4.16 ± 0.61	F=1.655	0.200
Blood platelet count	275.30 ± 97.21	272.80 ± 92.25	276.66 ± 100.10	F=0.075	0.784
Lymphocyte percentage	24.25 ± 9.03	24.88 ± 7.22	23.91 ± 9.88	F=0.545	0.461
Neutrophil absolute value	4.50 ± 1.82	4.28 ± 1.70	4.61 ± 1.88	F=1.572	0.211
Hemoglobin	117.16 ± 22.04	120.76 ± 13.97	115.22 ± 25.19	F=3.064	0.082
Total protein concentration	70.61 ± 6.59	71.78 ± 5.17	69.98 ± 7.18	F=3.650	0.057
Albumin levels	41.70 ± 4.95	43.56 ± 3.03	40.69 ± 5.48	F=17.415	<0.001

SD, standard deviation.

**Table 3** presents the correlations between sleep, social support, anxiety, depression, stress, and frailty in patients with gynecological cancers. The results showed significant positive correlations between frailty and sleep quality (r = 0.509), anxiety (r = 0.527), depression (r = 0.642), and stress (r = 0.658; all *p* < 0.001). By contrast, social support (PSSS) showed no significant correlation (r = 0.014, *p* = 0.843).

**Table 3:** Correlations between sleep, social support, anxiety, depression, and stress and frailty.

Characteristics	Aggregate score	
	r	P
PSSS	0.014	0.843
Sleep	0.509	<0.001
Anxiety	0.527	<0.001
Depression	0.642	<0.001
Stress	0.658	<0.001

PSSS, Perceived Social Support Scale.

In the final analysis, factors identified as significant in the univariate analysis were incorporated into the multivariate logistic regression model to determine their associations with frailty levels and compare their effects across frailty groups. The results revealed several factors associated with higher frailty levels, including older age (OR = 1.125, 95% CI:1.011–1.253), underweight status (OR = 0.037, 95% CI:0.002–0.664), stress (OR = 1.696, 95% CI:1.309–2.196), insomnia (OR = 1.300, 95% CI:1.033–1.636), married status (OR = 0.004, 95% CI:0.003–0.600), elevated white blood cell count (OR = 1.591, 95% CI:1.078–2.349), and low albumin levels (OR = 0.770, 95% CI:0.623–0.953). The results of the logistic regression analysis are summarized in **Table 4**.

**Table 4:** Logistic regression analysis of factors influencing frailty in gynecological malignancy patients undergoing surgery and chemotherapy.

Variable	β	SE	WaldX^2^	P	OR	95%CI
Age	0.118	0.055	4.644	0.031	1.125	1.011–1.253
Marital status (refer to: Married)			6.314	0.097		
Unmarried	-3.117	1.329	5.498	0.019	0.044	0.003–0.600
BMI (refer to: normal)			5.062	0.080		
Underweight	-3.301	1.476	5.004	0.025	0.037	0.002–0.664
Stress	0.528	0.132	16.014	<0.001	1.696	1.309–2.196
Insomnia	0.262	0.117	4.995	0.025	1.300	1.033–1.636
White blood count	0.464	0.199	5.459	0.019	1.591	1.078–2.349
Albumin concentration	-0.261	0.109	5.755	0.016	0.770	0.623–0.953

BMI, body mass index.

## 4 Discussion

This cross-sectional study had two important clinical findings. First, the prevalence of frailty in patients with gynecological malignancies who had undergone surgery and chemotherapy was 64.9%, substantially higher than the 42% median frailty prevalence reported in a systematic review of 2916 older patients with cancer from 20 studies in North America and Europe (29). The high prevalence of frailty in this study may be related to differences in patient population, treatment (surgery and chemotherapy), and frailty evaluation tools. Second, seven independent risk factors affecting frailty in patients undergoing surgery and chemotherapy were identified: older age, underweight status, stress, insomnia, married status, high white blood cell count, and low albumin levels.

Our findings demonstrated that older patients exhibited greater frailty, which is consistent with those of previous studies. Jauhari et al. conducted a cohort study in England and Wales, reporting that frailty prevalence rates in women aged 50–69, 70–79, and >80 years were 15%, 28%, and 47%, respectively (30). Age-related organ dysfunction and physiological decline may increase a patient’s vulnerability to stress and adverse health problems. These findings highlight the need for targeted frailty screening and early intervention in older patients with gynecological cancer undergoing surgery and chemotherapy.

Our findings indicated that the probability of frailty was significantly lower among unmarried patients than among married patients. This finding is consistent with an observational study in Italy, which reported that widows had a significantly lower risk of frailty than married women (31). Previous studies have indicated that marital status is closely related to cardiovascular diseases and diabetes (32–34) and may influence the progression of frailty by increasing the occurrence of these negative health issues. Additionally, post-menopausal women are more likely than men to face negative health problems, such as osteoporosis and abnormal blood glucose and lipid levels. These health issues are closely linked to accelerated frailty progression (35, 36), suggesting that the impact of marital status on frailty progression may be greater in women than in men. This study focuses on gynecological cancer patients, a specific population in which disease-treatment characteristics and social support patterns may moderate the impact of marital status on frailty. For instance, married patients may neglect their own rehabilitation needs due to caregiving responsibilities for children or spouses, or experience persistent psychological stress from concerns about the financial and emotional burden their illness places on the family, potentially exacerbating frailty. In contrast, unmarried patients may benefit from more flexible schedules to adhere to rehabilitation exercises and may also receive certain family social support, which could partly compensate for the absence of marital support. Therefore, medical institutions should conduct more cancer rehabilitation research that targets populations with different marital statuses and develop differentiated support strategies.

Our study found no significant correlation between high BMI and frailty but observed a significantly lower frailty risk among underweight patients compared to normal-weight patients. This conclusion differs from previous research findings (37, 38). Current studies predominantly discuss the pathophysiological mechanisms linking obesity and frailty, which include systemic inflammation, insulin resistance (IR), and oxidative stress (39). Urano et al. (40) also highlighted adiponectin, an important adipokine, as a potential biomarker for identifying frailty. However, our study found a statistically significant association between frailty and lower BMI, possibly because underweight individuals generally have lighter body weights, resulting in a lower basal metabolic burden and reduced energy expenditure, thereby delaying the onset of frailty. Moreover, obese or normal-weight individuals may experience chronic low-grade inflammation, whereas underweight individuals may have lower inflammation levels, potentially reducing the risk of frailty. Additionally, BMI classification standards do not differentiate between muscle and fat proportions. The finding that underweight patients appeared to have a lower risk of frailty should be interpreted cautiously, as it diverges from much of the existing literature. While one speculative explanation is that a subset of low-BMI individuals may have relatively preserved lean mass that supports function (41), this hypothesis cannot be confirmed with our data and warrants targeted assessment of body composition and muscle performance in future studies. These findings provide a new perspective on the prevention and management of frailty, and future research should explore the specific mechanisms linking body weight and frailty.

Our study identified stress as a significant risk factor for frailty among patients with gynecological tumors. This finding is noteworthy, as existing studies have primarily focused on the impact of negative emotions such as anxiety and depression on frailty (42, 43). Research has explained that older adults perceive frailty as a loss of autonomy and control, resulting in stress (44). A longitudinal study found that positive emotions reduced the risk of frailty (45). This can be explained by the stress-buffering model (46) and the “broadening and building” theory (47), in which positive emotions not only serve as a buffer against the negative effects of stress but also enable individuals to broaden their behavioral perspectives and abilities to draw on a wider range of physical, psychological, intellectual, and social resources to cope with stress. Therefore, it is important to regularly screen patients with cancer for frailty-related stress and manage patients at a high risk of stress in advance.

Our analysis showed that albumin levels were inversely associated with frailty. This has not been observed in other studies. Owing to the continuous depletion of cancer cells, patients with tumors are prone to hypoalbuminemia, which leads to skeletal muscle loss, decreased physical activity, and weakness (48). In addition, albumin is an important biochemical indicator of nutritional status (49, 50), and clinicians often use albumin levels to assess a patient’s risk of malnutrition. The results of this study also confirmed that poor nutritional status is an important risk factor for frailty. This highlights the importance of monitoring the albumin level and nutritional status of patients with tumors, conducting nutritional risk assessments, and increasing nutritional intake in time to improve their nutritional status and outcomes.

### 4.2 Limitations

This study has several limitations. First, the research subjects were restricted to patients who received combined surgery and chemotherapy. The prevalence of frailty in this specific population may be higher than that in the overall gynecological cancer patient population, which limits the generalizability of the study results. Second, this study did not conduct analyses for a single cancer type. The included multiple cancer types have inherent differences in clinical characteristics and disease trajectories, which may affect the observation of frailty distribution. Such heterogeneity may not only interfere with the results but also limit the ability to derive conclusions specific to particular cancer types. Future research will further stratify by tumor type on the basis of expanding the study population and, combined with comparisons of diverse treatment modalities, more accurately reveal the association between different cancer types and frailty. Third, the cross−sectional design limits this study to identifying associations rather than establishing causality. Future longitudinal cohort studies with expanded baseline data and long−term follow−up are needed to clarify the temporal and causal relationships between frailty and clinical factors. This study did not collect detailed comorbidity profiles (including type, severity, and duration), which limited our ability to comprehensively capture comorbidity−related characteristics that may influence the frailty status of patients with gynecological cancer. Comprehensive clinical information should be systematically collected, including detailed comorbidity profiles (type, severity, and duration), full medication records (including non−cancer drugs), specific chemotherapy regimens, treatment cycles, toxicity grades, and postoperative residual status. In addition, the sample size of this study is relatively limited, which may affect the robustness of the results. Furthermore, self-reported frailty assessment is susceptible to social desirability bias (e.g., gynecological cancer patients concealing symptoms due to privacy concerns) and comprehension difficulties among elderly patients (e.g., symptom matching deviations caused by cognitive decline), which may underestimate the prevalence of frailty. Future studies should adopt mixed measurement methods combining self-reports with objective indicators to mitigate such biases. Use of validated questionnaires with neutral prompts, and culturally sensitive wording can further reduce comprehension and desirability biases. Finally, the sample was drawn only from two tertiary hospitals in Shanghai, which may restrict the generalizability of the findings. As China’s economic center and a megacity, Shanghai possesses leading medical resources nationwide. Patients’ disease profiles, healthcare perceptions, and economic capacity may differ from those in medium or small cities. Therefore, caution is warranted when extending the conclusions to other regions. Future studies across diverse provinces, hospital tiers, and rural–urban contexts—and with measures of insurance status, socioeconomic indicators, and care pathways—are needed to assess transportability and strengthen external validity.

### 4.3 Conclusion

Frailty in patients with gynecological malignancies significantly influences cancer treatment decisions and outcomes. Timely interventions can help delay the progression from prefrailty to frailty, emphasizing the importance of early identification and management. This study reveals that factors such as older age, being underweight, stress, insomnia, married status, elevated white blood cell count, and low albumin levels are significant risk factors for frailty in patients with gynecological cancer undergoing surgery and chemotherapy. These findings provide valuable insights for identifying high-risk patients and designing targeted interventions to improve their quality of life and treatment outcomes. By addressing the modifiable risk factors, healthcare providers can develop personalized strategies to enhance care for this vulnerable population.

## Data Availability

The datasets used and analysed during the current study are available from the corresponding author or the first author on reasonable request. Requests for data can be directed to the corresponding author, Xia Duan (email: bamboo-714@163.com), or the first author, Tingting Wang (email: 18116427252@163.com).
